# Supplementary material for: A Single-Cycle Adenovirus Type 7 Vaccine for Prevention of Acute Respiratory Disease
Source: Viruses. 2019 May 3;11(5):413. doi: 10.3390/v11050413 (PMC6563269; doi:10.3390/v11050413)
Supplement: Supplementary file 1 [file viruses-11-00413-s001.pdf]

Supplementary Materials

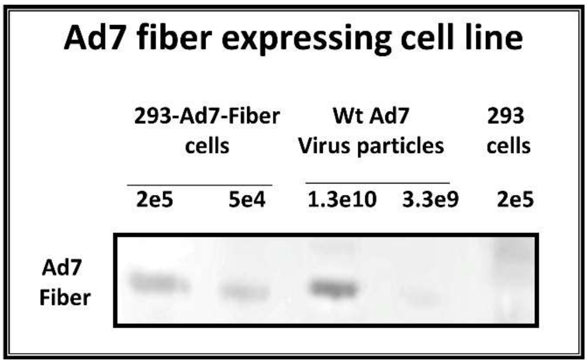

Figure S1. Ad7 Fiber Expressing cell line. We generated a cell line expressing codon-optimized Ad7 fiber as previously described [19]. A Western blot shows the Ad7 fiber protein expression by the 293-Ad7-fiber cell line as compared to wildtype Ad7 virus. This cell line was used to amplify the scAd7 expressing GFPLuc and DsRed.

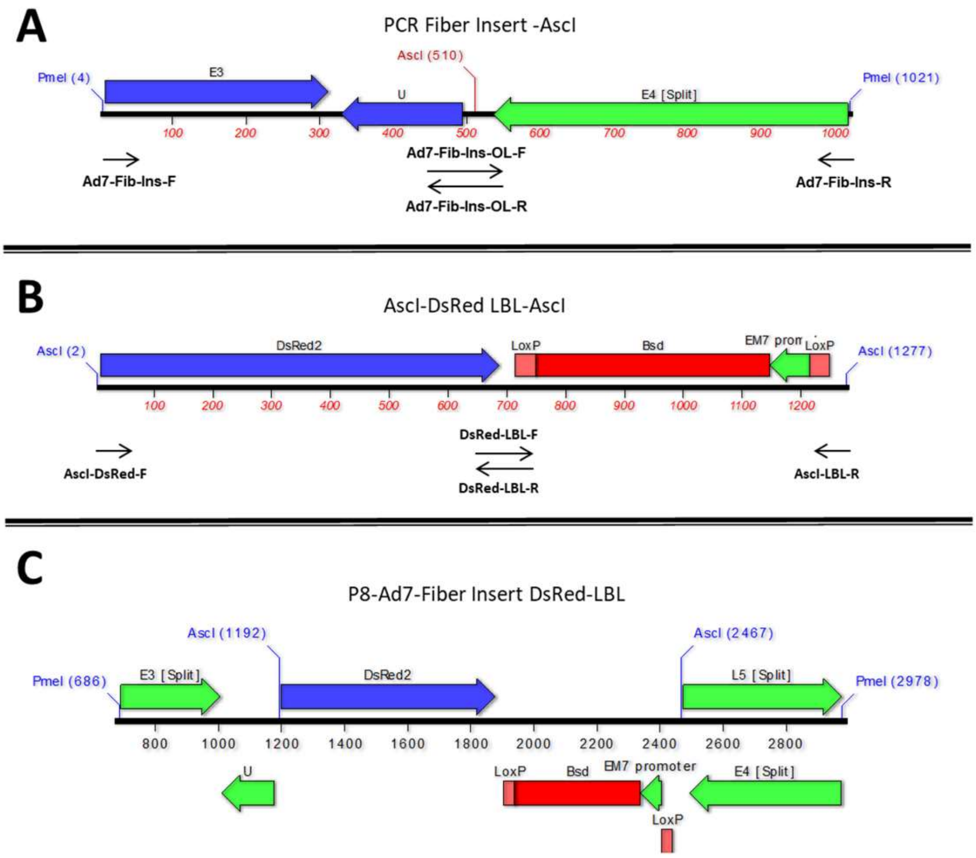

Figure S2. Overlapping PCR products used to create the scAd7 virus. In order to create the shuttle plasmid, 500 bp of fiber flanking DNA was PCR amplified by overlapping PCR. The PCR product was flanked by introduced PmeI sites and a unique cloning site, AscI, was introduced in between the flanking regions (**A**). The DsRed gene was fused to a Floxed blasticidin gene by overlapping PCR. The final PCR product contained 5′ and 3′ AscI sites for cloning into the shuttle plasmid (**B**). The final shuttle and DsRed insertion gene is shown (**C**). All PCR products were cloned into the Topo pCR8 cloning system and sequence verified.

Table S1. Infectious units of each virus as determined by FACS. The infectious units of each virus stock grown in 293 cells was determined by flow cytometry on A549 cells infected with serial dilutions of each virus. The percent GFP+ cells were determined after 48 h and used to calculate the FACS infectious units (IFU) per ml for each virus.

Virus	vp/mL	FACS IFU/mL	vp: IFU ratio
Ad7-dE1	3.5×10^{12}	1.88×10^{9}	1862:1
Ad7-dE3	1.8×10^{12}	5.58×10^{9}	322:1
scAd7	7.1×10^{12}	8.24×10^{9}	861:1
